# Supplementary material for: PIDDosome‐induced p53‐dependent ploidy restriction facilitates hepatocarcinogenesis
Source: EMBO Rep. 2020 Nov 23;21(12):e50893. doi: 10.15252/embr.202050893 (PMC7726793; doi:10.15252/embr.202050893)
Supplement: Supplementary file 1 — Appendix [file EMBR-21-e50893-s001.pdf]

Appendix Information

PIDDosome-induced p53-activation for ploidy restriction facilitates hepatocarcinogenesis

Valentina C. Sladky[1], Katja Knapp[1], Tamas G. Szabo[1], Vincent Z. Braun[1], Laura Bongiovanni[2], Hilda van den Bos[3], Diana C.J. Spierings[3], Bart Westendorp[2], Ana Curinha[4], Tatjana Stojakovic[5], Hubert Scharnagl[6], Gerald Timelthaler[7], Kaoru Tsuchia[8], Matthias Pinter[9], Georg Semmler[9], Floris Foijer[3], Alain de Bruin[2,10], Thomas Reiberger[9,11,12], Nataliya Rohr-Udilova[9], Andreas Villunger[1,11,12 #]

[1]Institute of Developmental Immunology, Biocenter, Medical University of Innsbruck, Innsbruck, AT;

[2]Department of Biomolecular Health Sciences, Faculty of Veterinary Medicine, Utrecht University, 3584 CL Utrecht, NL;

[3]European Research Institute for the Biology of Ageing, University of Groningen, University Medical Center Groningen, 9713 AV Groningen, NL;

[4]Institute of Pathophysiology, Biocenter, Medical University of Innsbruck, Innsbruck, AT;

[5]University Hospital Graz, Clinical Institute of Medical and Chemical Laboratory Diagnostics, Graz AT,

[6]Medical University of Graz, Clinical Institute of Medical and Chemical Laboratory Diagnostics, 8010 Graz, AT;

[7]Institute for Cancer Research, Internal Medicine I, Medical University of Vienna, 1090 Vienna, AT;

[8]Musashino Red Cross Hospital, Dept. of Gastroenterology & Hepatology, Tokyo, Japan,

[9]Division of Gastroenterology and Hepatology, Department of Medicine III, Medical University of Vienna, 1090 Vienna, AT;

[10]Department Pediatrics, University Medical Center Groningen, University Groningen, 9713 AV Groningen, NL

[11]Ludwig Boltzmann Institute for Rare and Undiagnosed Diseases (LBI-RUD), 1090 Vienna, AT;

[12]CeMM Research Center for Molecular Medicine of the Austrian Academy of Sciences, 1090 Vienna, AT.

Table of content

Appendix table S1 – p.1

Appendix table S2 – p.2

Appendix tables

Parameter	Overall cohort (n=223)	Low tumor density (high ploidy; n=111*)	High tumor density (low ploidy; n=111*)	P value
**Age (years)**	58.2±10.0	58.0±9.4	58.3±10.6	0.831
**Sex**				
Male	197 (88.3%)	101 (91.0%)	95 (85.6%)	0.210
Female	26 (11.7%)	10 (9.0%)	16 (14.4%)	
**Tumor size (cm)^{X}**	3.84±3.56	3.60±3.82	4.09±3.24	0.367
**Vascular invasion^{X}**				
Yes	15 (8.7%)	2 (2.2%)	12 (15.2%)	**0.002**
No	157 (91.3%)	90 (97.8%)	67 (84.8%)	
**Number of nodules^{X}**				
1	60 (34.9%)	36 (39.1%)	23 (29.1%)	0.332
2	38 (22.1%)	16 (17.4%)	22 (27.8%)	
3	47 (27.3%)	26 (28.3%)	21 (26.6%)	
≥4	27 (15.7%)	14 (15.2%)	13 (16.5%)	
**Etiology**				
ALD	65 (29.1%)	39 (35.1%)	25 (22.5%)	0.074
HBV	24 (10.8%)	15 (13.5%)	9 (8.1%)	
HCV	85 (38.1%)	38 (34.2%)	47 (42.3%)	
NASH	33 (14.8%)	14 (12.6%)	19 (17.1%)	
Other	16 (7.2%)	5 (4.5%)	11 (9.9%)	

Appendix table S1: Clinical characteristics of patients included in recurrence-free survival and cell density analysis. Data on tumor size and vascular invasion were not available for all patients. Those were excluded from the multivariate analysis. * One patient with exactly the median tumor density was not included in the comparisonn. ^{X} Data were available in 172 patients. Data are presented as relative percentages. Data are represented as mean ± STD.

**Risk factor for recurrence after transplantation**	**Adjusted Hazard Ratio (95% confidence interval)**	**Significance level after backward elimination**
**HCC cell density (low ploidy)**	**1.30 (95% CI: 1.05-1.62)**	**p = 0.004**
**HCC Tumor diameter (per cm)**	**1.11 (95% CI: 1.04-1.19)**	**p = 0.021**
Vascular invasion (present vs. absent)	1.11 (95% CI: 0.44-2.80)	p = 0.820
Sex (male vs. female)	0.91 (95% CI: 0.43-1.94)	p = 0.809
Age (per year)	1.00 (95% CI: 0.97-1.03)	p = 0.898

Appendix table S2: Multivariate analysis to evaluate the impact of tumor cell density as independent parameter on recurrence-free survival.
