# Supplementary material for: PIDDosome‐induced p53‐dependent ploidy restriction facilitates hepatocarcinogenesis
Source: EMBO Rep. 2020 Nov 23;21(12):e50893. doi: 10.15252/embr.202050893 (PMC7726793; doi:10.15252/embr.202050893)
Supplement: Supplementary file 2 — Expanded View Figures PDF [file EMBR-21-e50893-s002.pdf]

Expanded View Figures

Figure EV1. Characterization of DEN-induced liver cancers in PIDDosome mutant mice.

A Representative overviews of tumor-bearing murine liver tissue of all genotypes used. *N*-numbers refer to biological replicates. Scale bar size equals 540 μm.
B, C Histopathological analysis was performed to define tumor classification and associated and pre-neoplastic lesions in DEN-treated mice.
D Levels of serum parameters indicative for liver function and integrity in all genotypes tested. AST, aspartate aminotransferase; ALT, alanine aminotransferase. wt $n = 21$, *Casp2*$^{-/-}$ $n = 11$, *Raidd*$^{-/-}$ $n = 12$, *Pidd1*$^{-/-}$ $n = 11$.
E The grade of inflammation was histopathologically analyzed in tumorous (T) and non-tumorous (NT) tissue of HCC bearing DEN-treated mice. The central band represents the median, the boxes indicate the 25th and the 75th percentile, and the whiskers represent the range.
F The type of immune infiltrate in DEN-induced HCC tumor tissue was determined as % incidence. Infiltrated found were leukocytes (L), neutrophils (N), plasma cells (P), or mixed population (L + N; L + P + N). No macrophages were found in the immune infiltrates.
G Size distribution of DEN-induced surface tumors, data are shown as mean ± SEM.

Data information: Number of animals examined in (B, C, E–G): T and NT: wt $n = 18$, *Casp2*$^{-/-}$ $n = 12$, *Raidd*$^{-/-}$ $n = 14$, Pidd1$^{-/-}$ $n = 15$. Data are represented as median; statistical significance was determined by one-way ANOVA with multiplicity correction (Sidak–Holm); *$P \leq 0.05$, **$P \leq 0.01$.

© 2020 The Authors

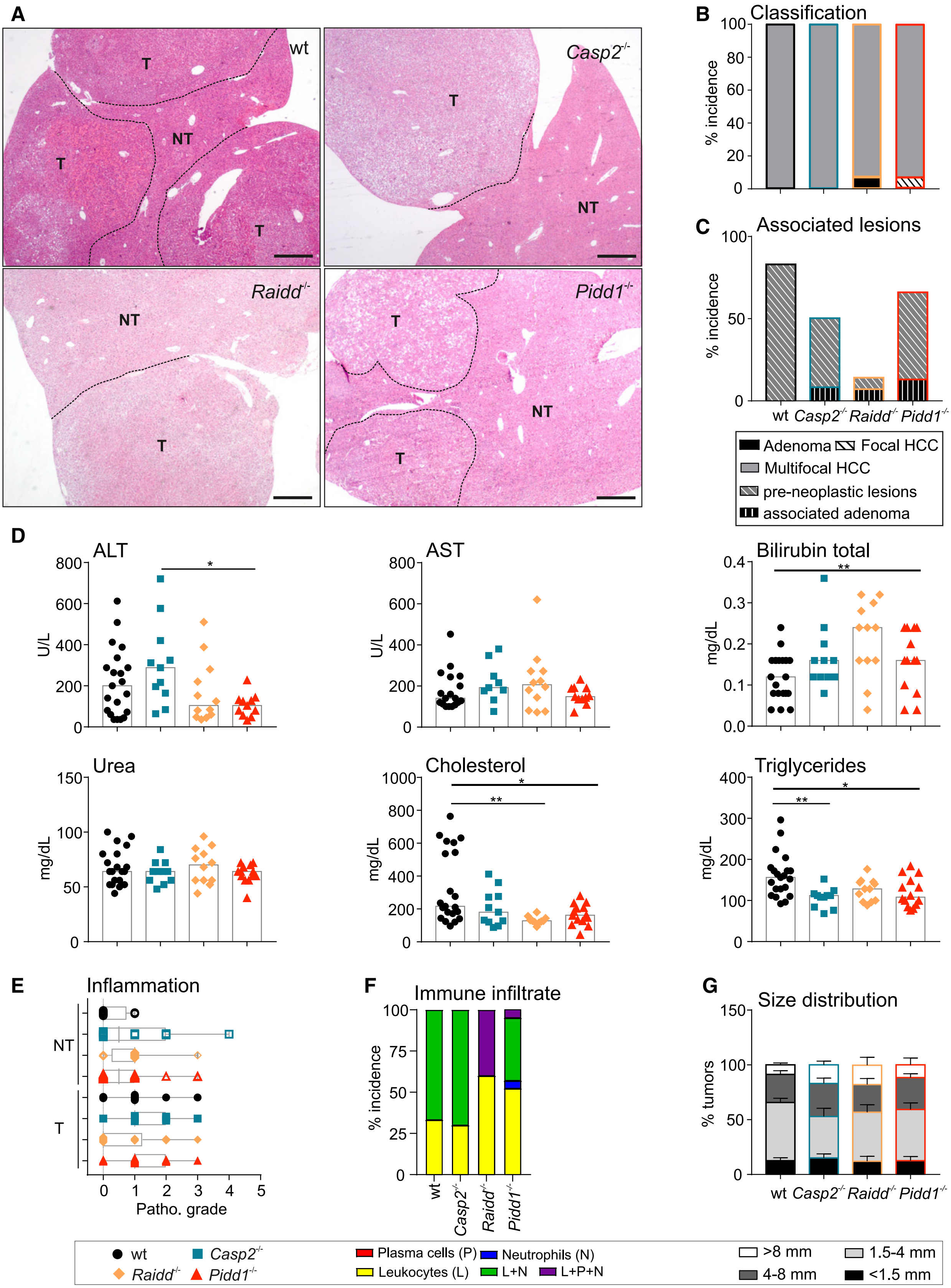

Figure EV1.

 © 2020 The Authors

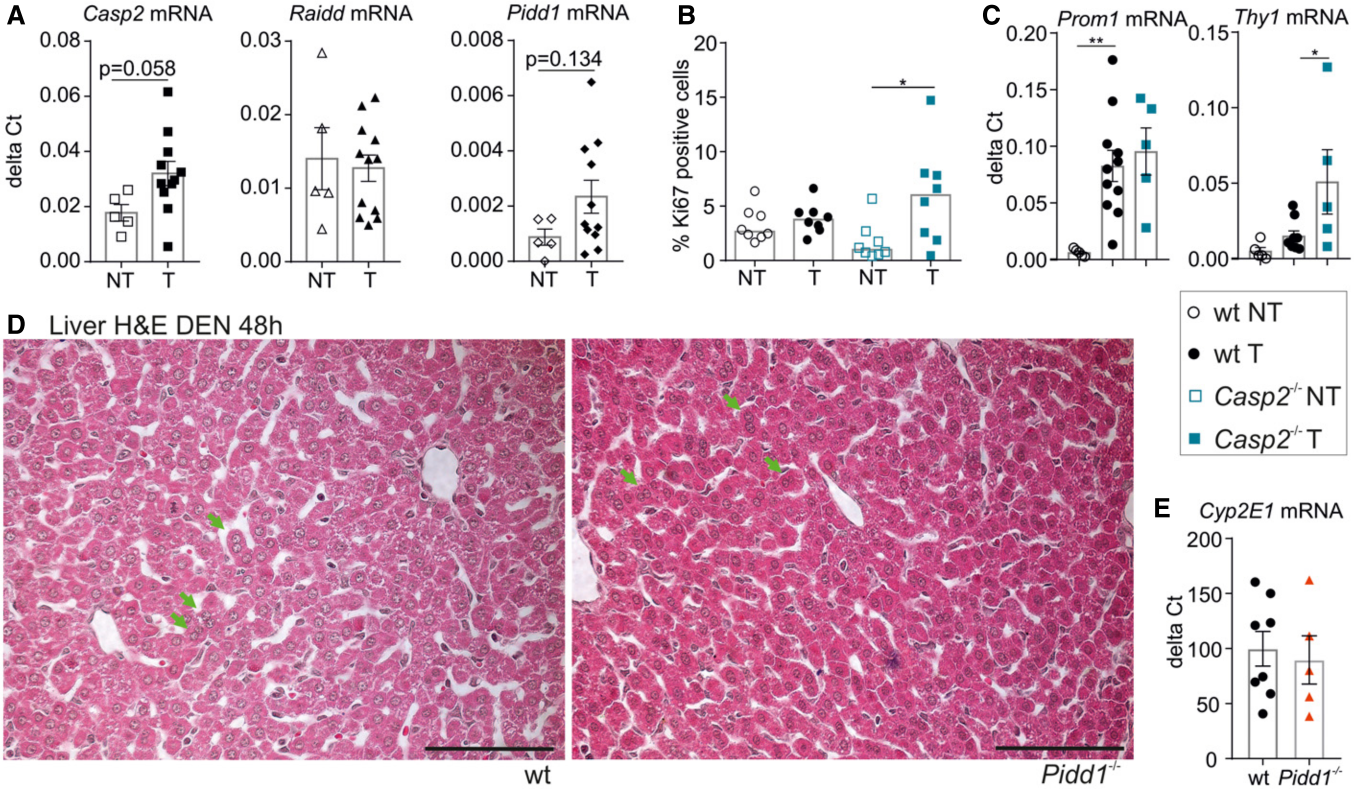

Figure EV2. Molecular characterization of PIDDosome-deficient liver cancer specimens.

A Transcript levels of *Casp2*, *Raidd* ($P = 0.74$) and *Pidd1* in non-tumorous (NT) and tumor tissue (T) from mice bearing DEN-induced HCC. *Hprt* mRNA expression was used for normalization.

B Proliferation was assessed by immunostaining of Ki67 on paraffin-embedded tissue sections; $n = 8$ per genotype and tissue. Data are shown as median.

C Transcript levels of Prom1 and Thy1, the genes encoding for the cancer stem cell markers CD133 and CD90, in NT and T of wt and T of *Casp2*$^{-/-}$ mice with DEN-HCC. The Ct values were normalized over Hprt expression.

D Representative images of H&E-stained liver sections that were used for the quantification of binucleated cells in Fig 2E (green arrows mark examples). Scale bar represents 100 μm.

E Transcript levels of Cyp2E1 in wt ($n = 8$) and *Pidd1*$^{-/-}$ ($n = 5$) livers from 15-day-old mice.

Data information: Data in (A, C, E) are represented as mean ± SEM from independent biological replicates. Statistical significance was determined by unpaired, two-tailed Student's *t*-test or one-way ANOVA with Sidak–Holm correction for multiplicity (C); $^{*}P \leq 0.05$, $^{**}P \leq 0.01$. *N*-numbers refer to biological replicates.

© 2020 The Authors

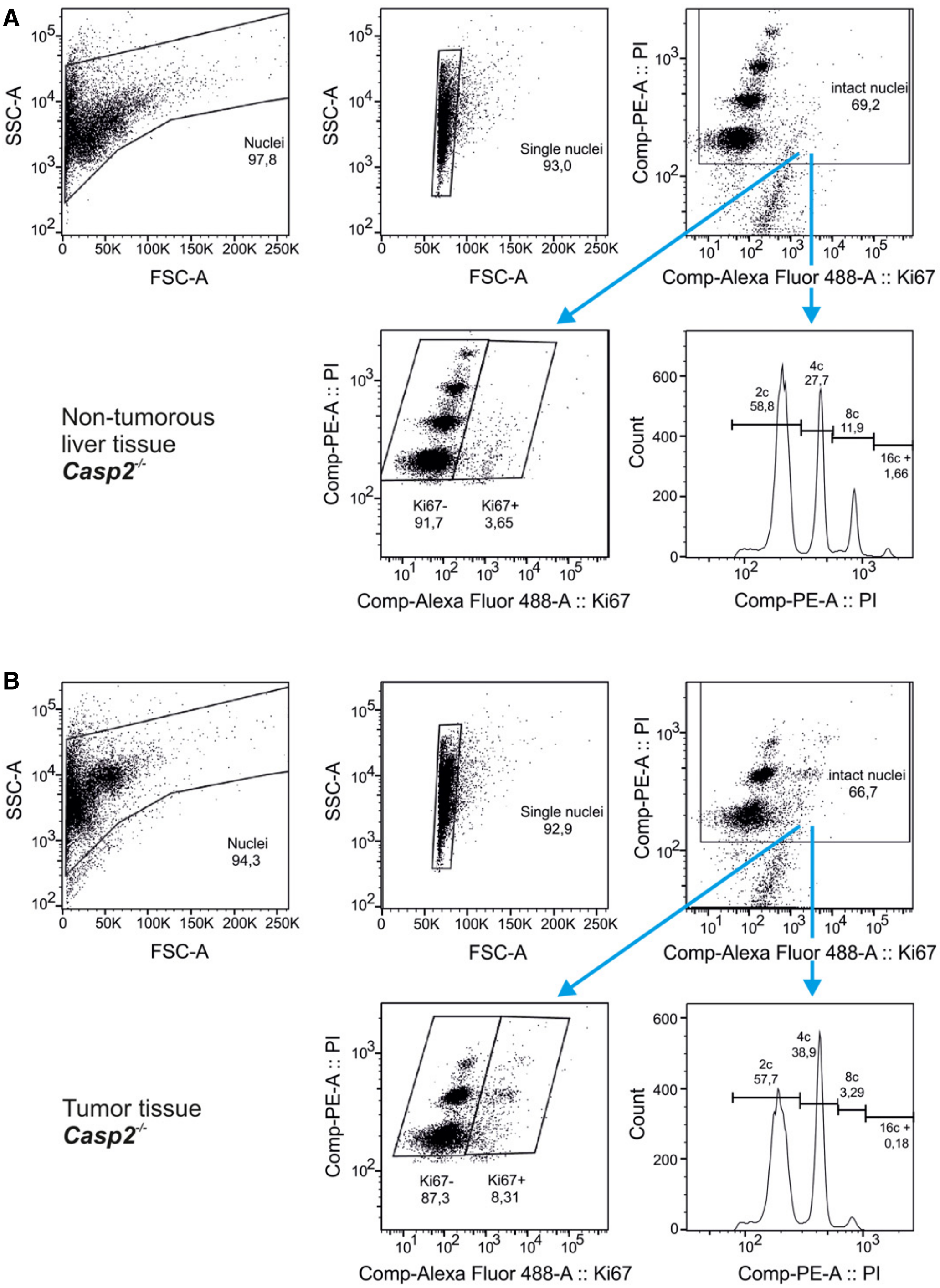

Figure EV3. Ploidy analysis of liver tumor specimens by flow cytometry.

A, B Gating strategy for DNA content analysis using flow cytometry to determine the ploidy distribution of non-tumorous (A) and tumor tissue (B).

 © 2020 The Authors

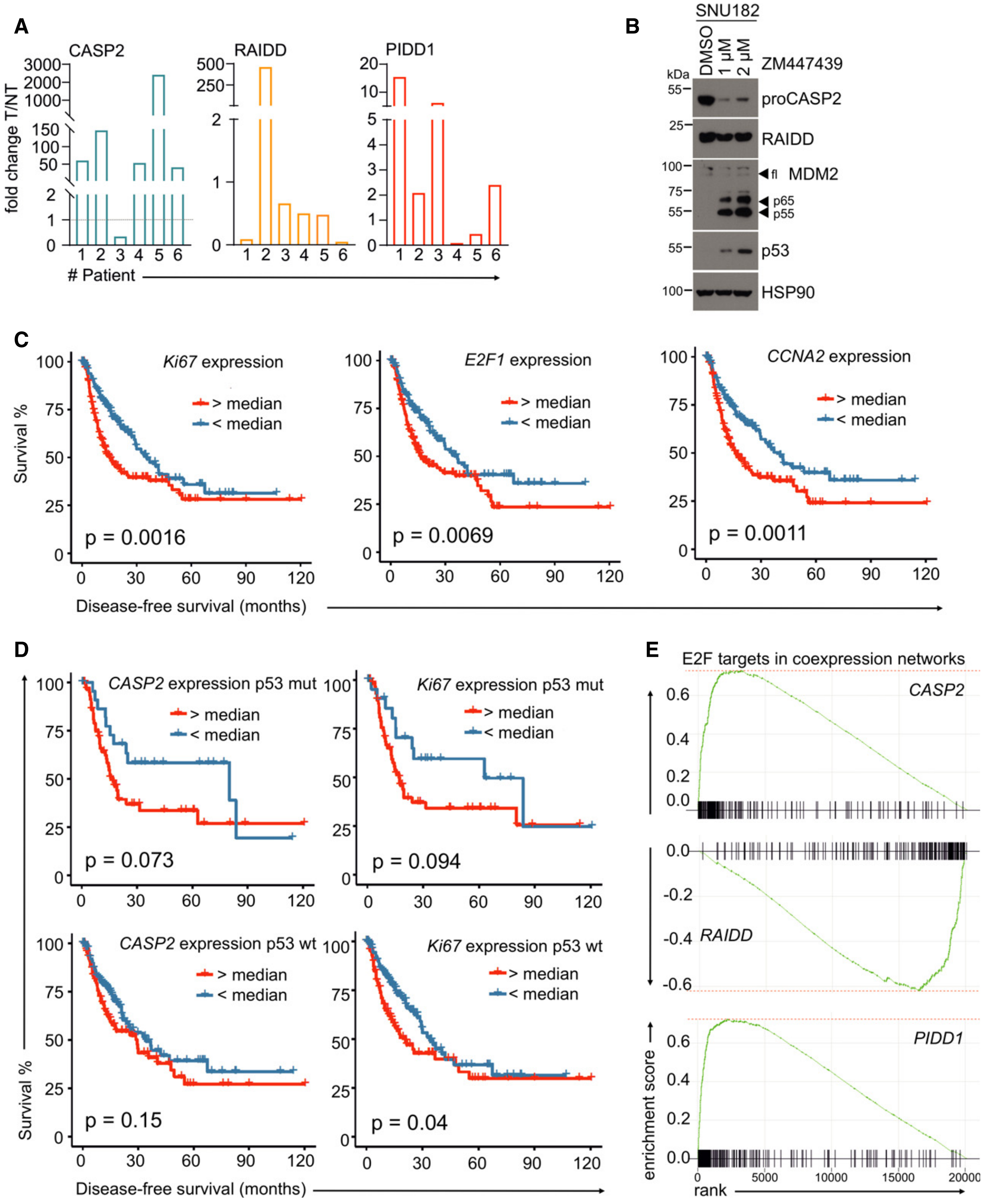
A
CASP2
RAIDD
PIDD1
fold change T/NT
Patient
B
SNU182
DMSO
1 μM
2 μM
ZM447439
kDa
proCASP2
RAIDD
MDM2
fl
p65
p55
p53
HSP90
C
Ki67 expression
E2F1 expression
CCNA2 expression
> median
< median
Survival %
p = 0.0016
p = 0.0069
p = 0.0011
Disease-free survival (months)
D
CASP2 expression p53 mut
Ki67 expression p53 mut
p = 0.073
p = 0.094
CASP2 expression p53 wt
Ki67 expression p53 wt
p = 0.15
p = 0.04
E
E2F targets in coexpression networks
CASP2
RAIDD
PIDD1
enrichment score
rank

Figure EV4.

Figure EV4. Evaluation of PIDDosome expression levels and its prognostic value in human HCC.

A Densitometric quantification of the immunoblot in Fig 5A is shown as the fold change in tumor (T) tissue over non-tumorous (NT) tissue of the individual patients. The values for proCASP2, PIDD-C, and RAIDD were normalized to the corresponding PonceauS staining.

B Immunoblot of the HCC cell line SNU182 treated with DMSO or increasing concentrations of the Aurora B inhibitor ZM447439. The membrane was probed for CASP2, RAIDD, MDM2, p53, and HSP90 as loading control.

C Recurrence-free survival of HCC patients in the TGCA Provisional data set (LIHC) expressing high or low levels of the proliferation-associated genes *Ki67*, *E2F1*, and *CCNA2*.

D Disease-free survival of the TGCA HCC patients shown in Fig 5C was analyzed with respect to the p53 (mutated or wt) status. Data are divided at the median, and statistical significance was tested using a log-rank test.

E Butterfly plots showing the enrichment of E2F target genes in the co-expression networks of *CASP2*, *RAIDD*, and *PIDD1* in the same TCGA data set.

Source data are available online for this figure.

Figure EV5. Evaluating nuclear size as a predictor of HCC outcome.

A A subset of 39 HCC patient images was analyzed before and after introducing a circularity cutoff (37 HCV, 2 HBV patients). Nuclei above a cutoff of 7 out of 10 were considered as hepatocytes or tumor cells.

B, C Representative examples of the morphometric analyses of tumor biopsies from HCC patients with HCV (B) or ALD (C). Paraffin-embedded tissue sections were stained with hematoxylin and tryptase. Tissue Studio® allows analysis of the cell density by detection of nuclei (blue) and cell area (green) and can be used to classify the cells by size as indicated by the color code. Scale bar size equals 200 μm.

© 2020 The Authors

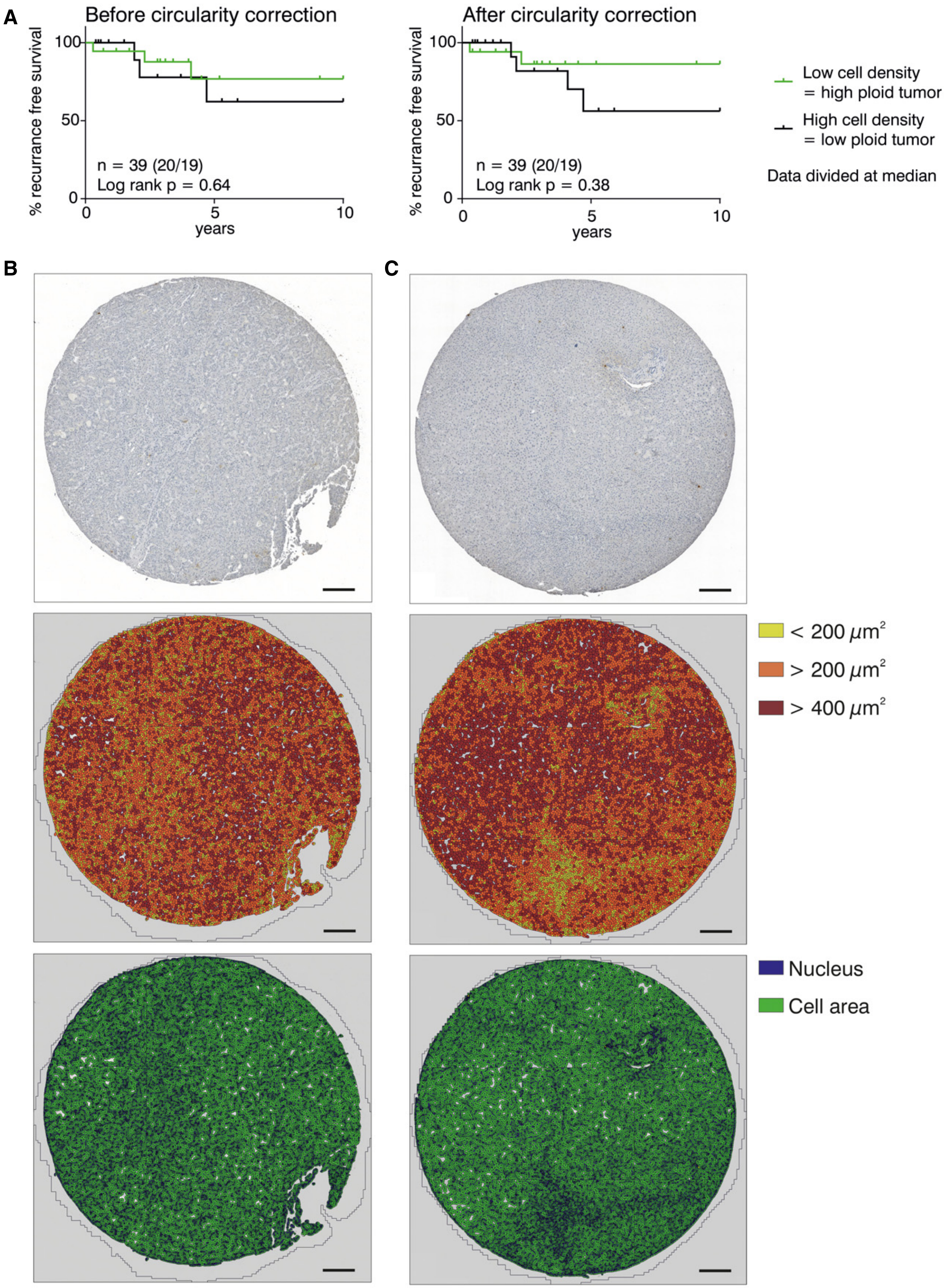
A
Before circularity correction
% recurrance free survival
100
50
0
n = 39 (20/19)
Log rank p = 0.64
0
5
10
years
After circularity correction
% recurrance free survival
100
50
0
n = 39 (20/19)
Log rank p = 0.38
0
5
10
years
Low cell density
= high ploid tumor
High cell density
= low ploid tumor
Data divided at median
B
C
< 200 µm²
> 200 µm²
> 400 µm²
Nucleus
Cell area

Figure EV5.
