# Supplementary figures and images for: PIDDosome‐induced p53‐dependent ploidy restriction facilitates hepatocarcinogenesis
Source: EMBO Rep. 2020 Nov 23;21(12):e50893. doi: 10.15252/embr.202050893 (PMC7726793; doi:10.15252/embr.202050893)

Figure EV4B

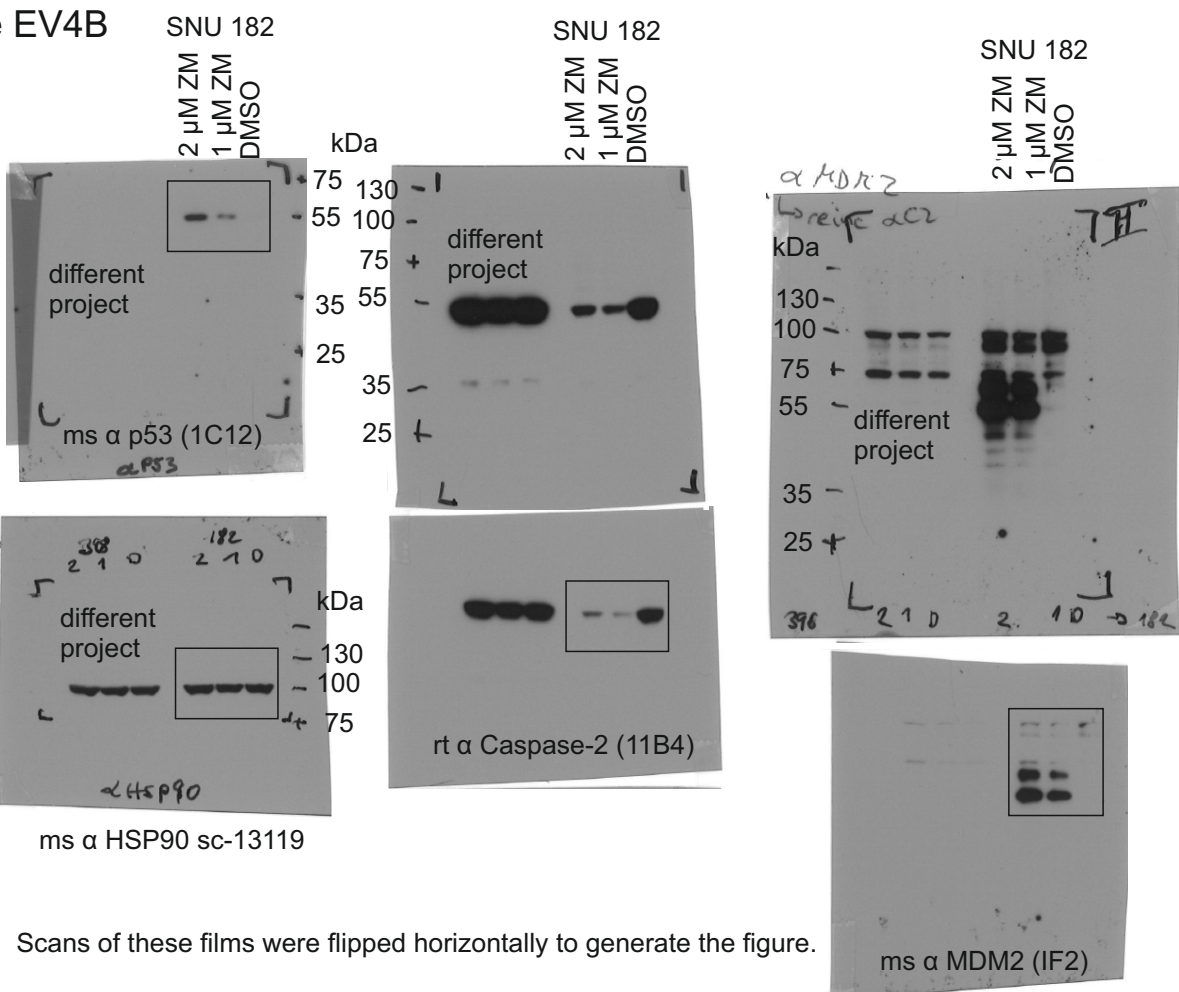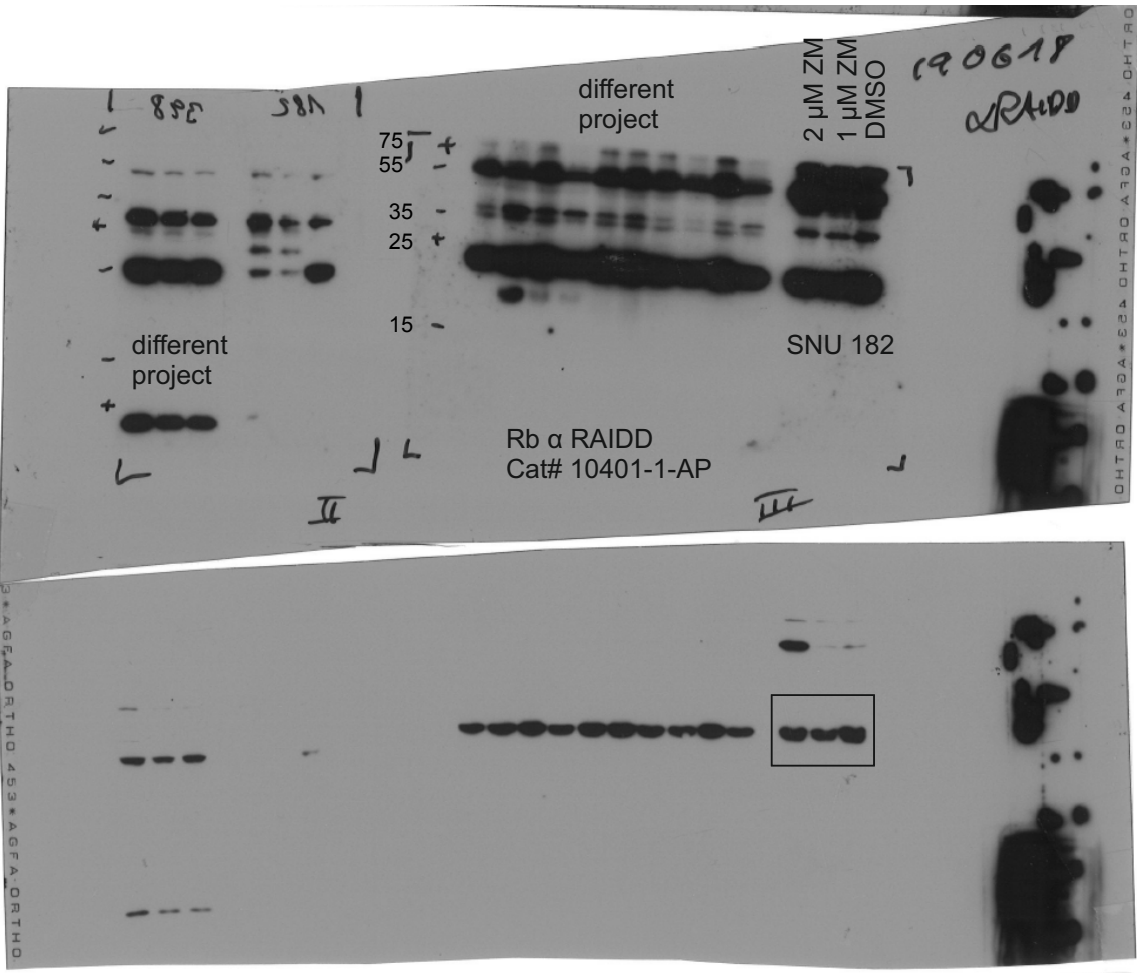

Supplement: Supplementary file 3 — Source Data for Expanded View [file EMBR-21-e50893-s005.pdf]

Figure 2A

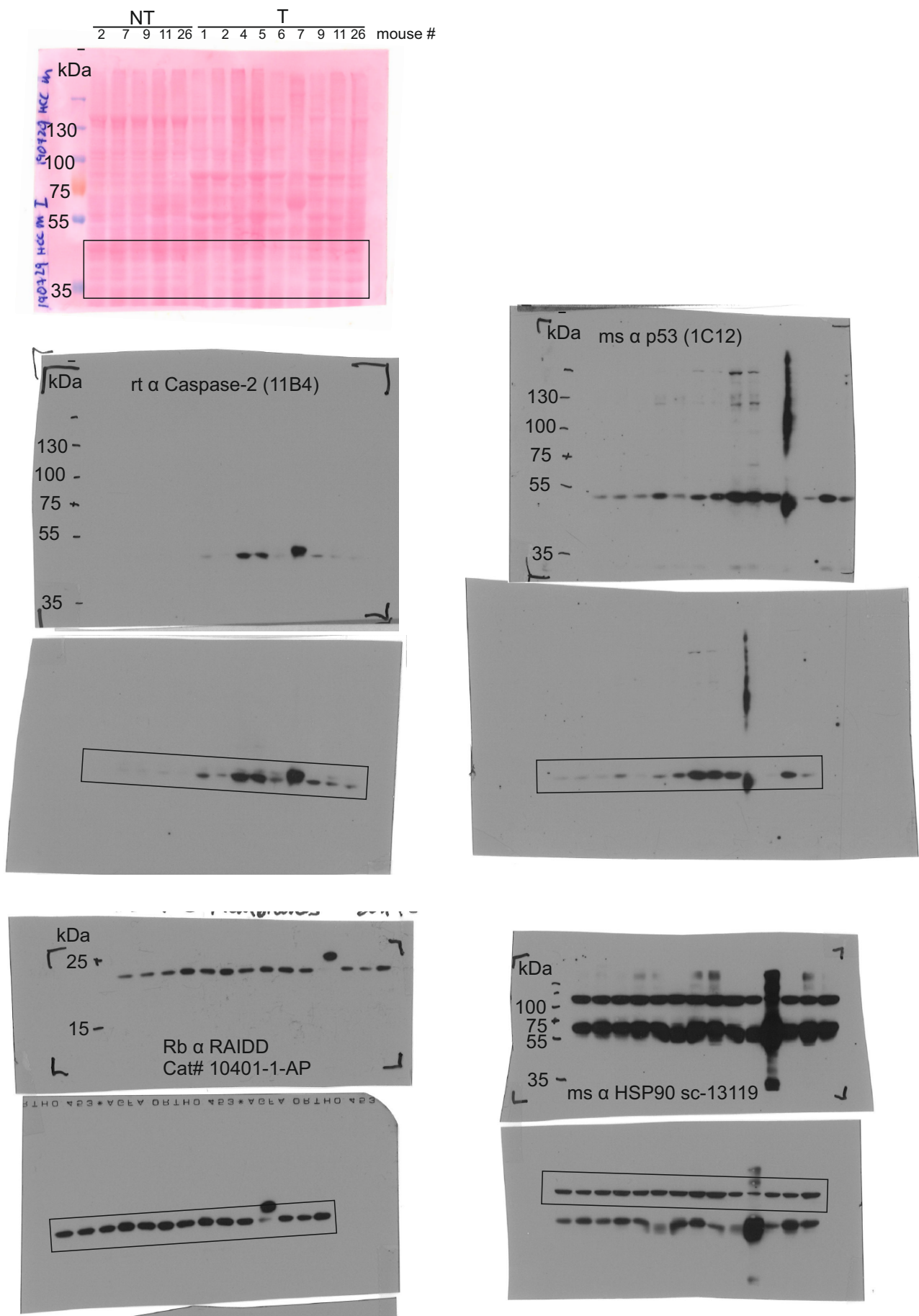

Figure 2D

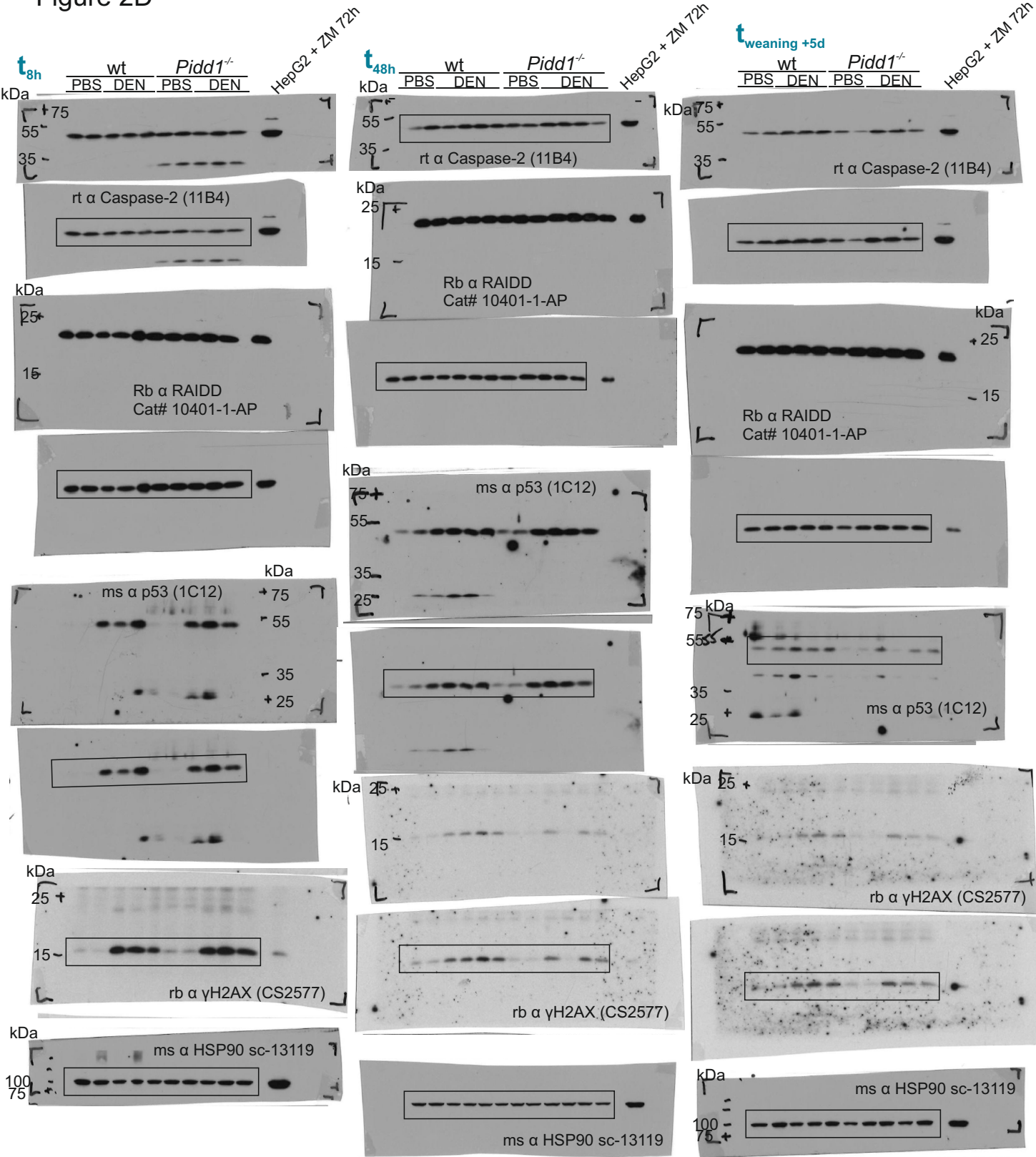

Supplement: Supplementary file 5 — Source Data for Figure 2 [file EMBR-21-e50893-s003.pdf]

Figure 5A

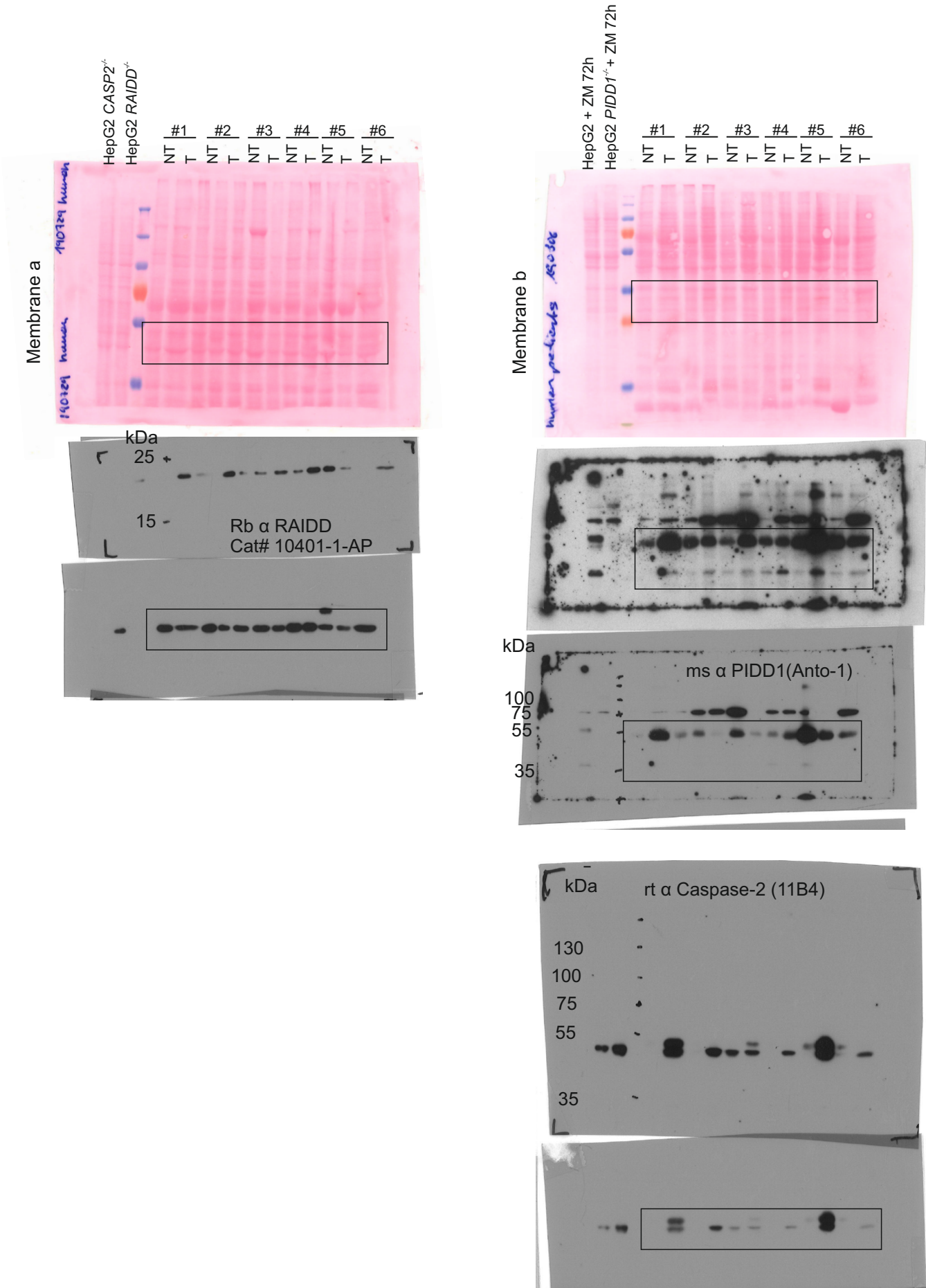

Supplement: Supplementary file 6 — Source Data for Figure 5 [file EMBR-21-e50893-s004.pdf]
